# GREM1 signaling in cancer: tumor promotor and suppressor?

**DOI:** 10.1007/s12079-023-00777-4

**Published:** 2023-08-24

**Authors:** Zhichun Gao, Julia M. Houthuijzen, Peter ten Dijke, Derek P. Brazil

**Affiliations:** 1 Wellcome-Wolfson Institute for Experimental Medicine, Queen’s University Belfast, 97 Lisburn Road, Northern Ireland, BT9 7BL UK; 2 Oncode Institute, Division of Molecular Pathology, Netherlands Cancer Institute, Plesmanlaan 121, Amsterdam, 1066 CX The Netherlands; 3 Oncode Institute, Department of Cell and Chemical Biology, Leiden University Medical Center, Leiden, The Netherlands

## Abstract

**Abstract:**

GREMLIN1 (GREM1) is member of a family of structurally and functionally related secreted cysteine knot proteins, which act to sequester and inhibit the action of multifunctional bone morphogenetic proteins (BMPs). GREM1 binds directly to BMP dimers, thereby preventing BMP-mediated activation of BMP type I and type II receptors. Multiple reports identify the overexpression of GREM1 as a contributing factor in a broad range of cancers. Additionally, the *GREM1* gene is amplified in a rare autosomal dominant inherited form of colorectal cancer. The inhibitory effects of GREM1 on BMP signaling have been linked to these tumor-promoting effects, including facilitating cancer cell stemness and the activation of cancer-associated fibroblasts. Moreover, GREM1 has been described to bind and signal to vascular endothelial growth factor receptor (VEGFR) and stimulate angiogenesis, as well as epidermal and fibroblast growth factor receptor (EGFR and FGFR) to elicit tumor-promoting effects in breast and prostate cancer, respectively. In contrast, a 2022 report revealed that GREM1 can promote an epithelial state in pancreatic cancers, thereby inhibiting pancreatic tumor growth and metastasis. In this commentary, we will review these disparate findings and attempt to provide clarity around the role of GREM1 signaling in cancer.

**Graphical Abstract:**

**Figure:**
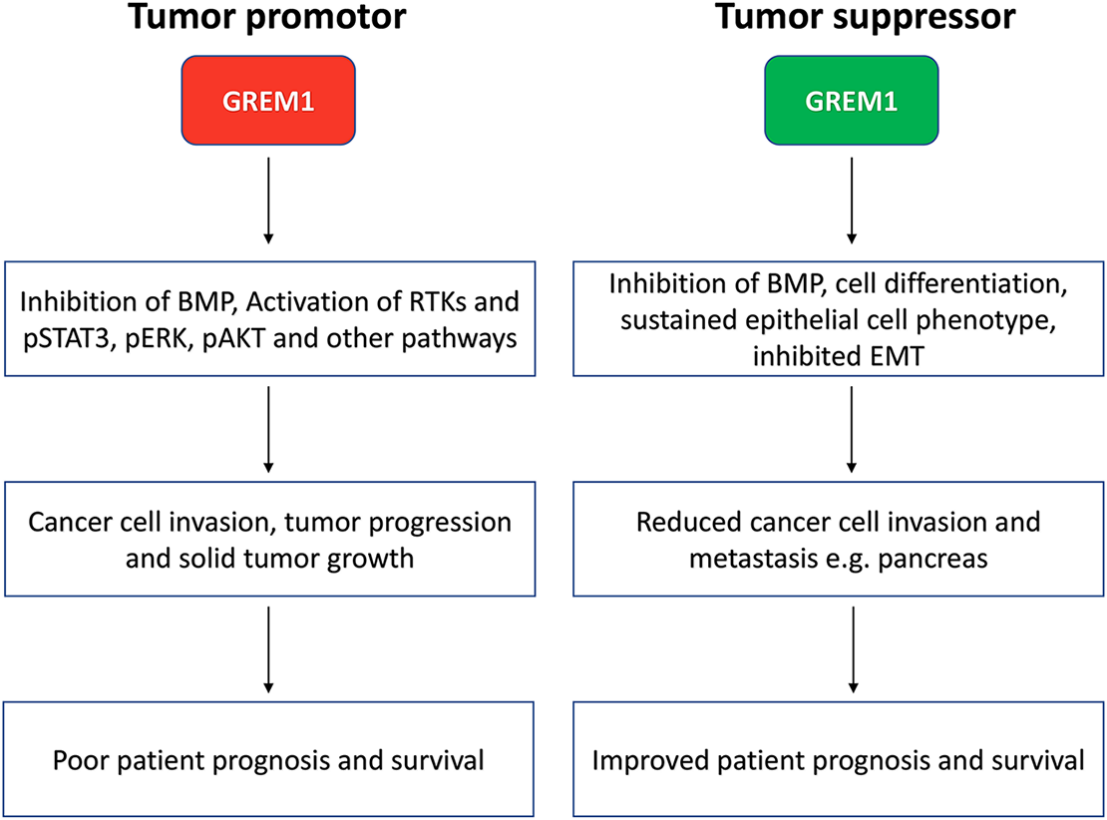

## Introduction

**Fig. 1:**
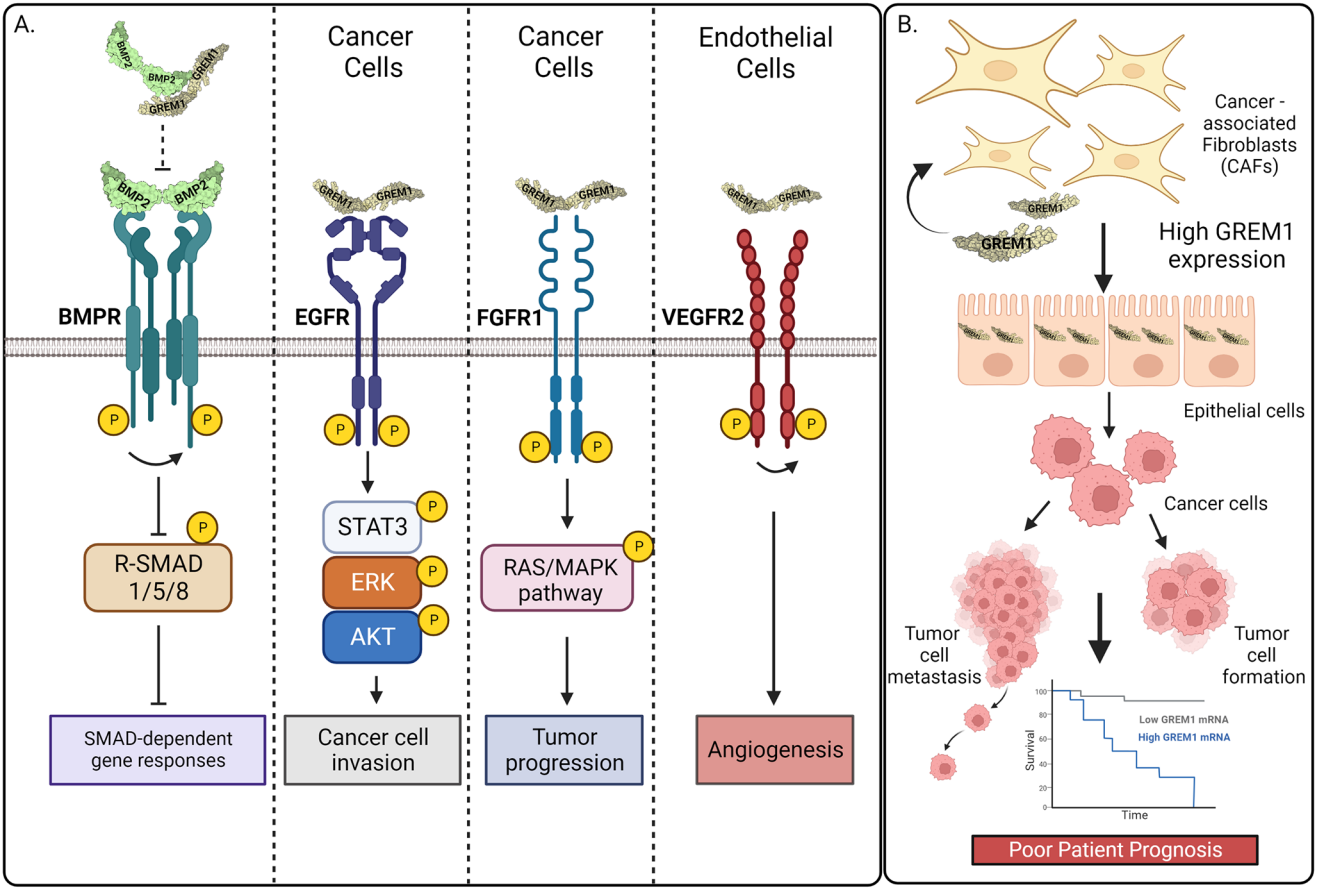
Proposed mechanisms of GREM1 signaling in cancer: Bad Cop/Good Cop. **A** Canonical GREM1 signaling involves GREM1 dimers binding to BMP dimers to prevent BMPR activation and receptor R-SMAD1/5/8 signaling. GREM1 binding to EGFR leads to phosphorylation and activation of STAT3, ERK and AKT in breast cancer cells, facilitating increased invasion (Kim et al. 2020). GREM1 dimer binding to FGFR1 leads to activation of RAS/ERK signaling and in androgen-insensitive prostate cancer cells, driving tumor progression (Lan et al. 2022). GREM1 binding to VEGFR2 has been postulated to drive VEGFR2 activation in endothelial cells, leading to angiogenesis (Mitola et al. 2010). **B** Increased *GREM1* expression from specific subsets of CAFs leads to epithelial-mesenchymal transition and the formation of mesenchymal cancer cells, increased solid tumor formation and tumor cell invasion and metastasis, all of which contribute to poorer patient prognosis in colorectal and other cancers. *GREM1* also has an autocrine effect on CAFs, indicated by the curved arrow. Kaplan Meier curve is illustrative of improved survival of patients with low *GREM1* mRNA in tumor biopsies (*grey line*) versus lower survival where GREM1 mRNA are higher (*blue line*). **C** Increased methylation of GREM1 promotor regions and reduced *GREM1* mRNA were detected in renal cell carcinoma (Morris et al. 2010). **D** High levels of *GREM1* mRNA in stromal fibroblasts from CRC were associated with improved patient survival (Jang et al. 2017). **E** High GREM1 protein expression in pancreatic NETs correlated with high microvessel density and increased NET differentiation, as well as improved recurrence-free patient survival (Chen et al. 2013). **F** *Grem1* secretion from pancreatic tumor cells that have undergone EMT is proposed to act on pancreatic epithelial cells to inhibit pro-EMT Snail and Slug transcription factor expression and maintain the undifferentiated epithelial cell phenotype in the tumor compartment (Lan et al. 2022). For Panel **B** and **D**, Kaplan Meier curves are illustrative of improved survival of patients with high *GREM1* mRNA levels in tumor biopsies (*grey line*) versus lower survival where *GREM1* mRNA are lower (*blue line*). Figures created with BioRender.com

GREMLIN1 (GREM1) is a secreted member of the DAN family of cysteine knot proteins that binds to and inhibits the actions of bone morphogenetic proteins (BMPs) such as BMP2 and BMP4 (Todd et al. 2020; Gomez-Puerto et al. 2019; Mulloy and Rider, 2015). BMPs signal via heteromeric complexes of specific transmembrane BMP type II and type I serine threonine kinase receptors (BMPRII and BMPRI) and intracellular SMAD transcription factors (Sanchez-Duffhues et al. 2020). Binding of GREM1 to BMPs was suggested to form fibril-like oligomeric complex that prevents BMP binding to its cognate BMPRII/BMPRI heterotetrameric complex, thus attenuating BMP signaling (Kišonaitė et al. 2016) (Fig. 1A). An exquisite balance of GREM1/BMP signaling is required for a range of developmental processes such as kidney and digit formation. *Grem1*-/- mice display defective lower limb and digit formation, and the majority die 1–2 days after birth due to renal agenesis (Khokha et al. 2003; Michos et al. 2004). Importantly, renal agenesis in *Grem1*-/- mice was rescued by the deletion of one allele of *Bmp4* (Michos et al. 2007). The normal kidney development of *Grem1*-/-;*Bmp4*+/- mice emphasised that a normal, physiological “volume” of *Bmp4* signaling is required, and perturbations that either decrease *Grem1* signaling or increase *Bmp4* signaling can have dramatic effects on organ and tissue formation. This idea is supported by several mouse models of *Grem1* transgenic overexpression or genetic loss of *Grem1* in bone (Worthley et al. 2015; Gazzerro et al. 2007; Gazzerro et al. 2005). A feedback loop involving Grem1, Fgf4 and sonic hedgehog (Shh) has been shown to be critical for mouse limb development (Khokha et al. 2003). For kidney development, Grem1-mediated inhibition of Bmp4 facilitates the establishment of an autoregulatory Gdnf/Wnt11 feedback network that enables ureteric bud outgrowth (Michos et al. 2007). Therefore, tight spatiotemporal signaling between GREM1, BMPs and other signaling pathways is essential for normal mammalian development and healthy tissue function.

**Figure:**
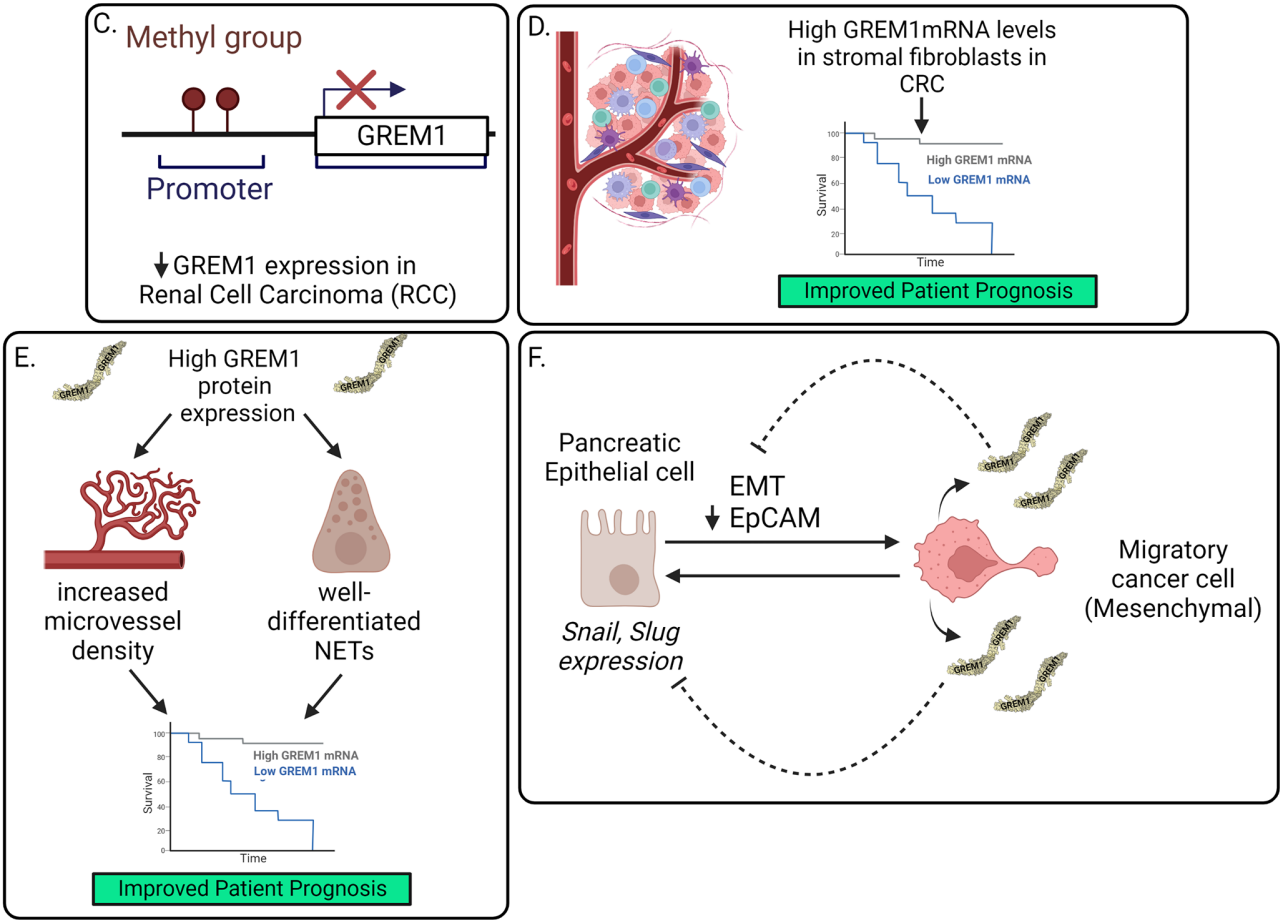

In addition to BMPs, GREM1 has been reported to bind to other soluble proteins and interact with several receptor tyrosine kinases (RTKs), including vascular endothelial growth factor receptor (VEGFR) supporting angiogenesis and epidermal growth factor receptor (EGFR) (Mitola et al. 2010; Park et al. 2020) and most recently fibroblast growth factor receptor (FGFR1) (Cheng et al. 2022) promoting cancer progression (Fig. 1A). In contrast, a report recently emerged that GREM1 signaling contributes to inhibition of pancreatic tumor growth and cancer progression (Lan et al. 2022). This commentary will briefly summarize the data on GREM1 binding proteins, as well as putative mechanisms of GREM1-mediated pathogenesis in cancer. We will then discuss the considerably fewer number of reports in which GREM1 is described to act as a tumor suppressor.

## GREM1 biology

Originally identified as a pathogenic mediator of diabetic nephropathy (DN) (Dolan et al. 2005), amplified *GREM1* mRNA levels have also been implicated in idiopathic pulmonary fibrosis (Myllärniemi et al. 2008), pulmonary artery hypertension (Wellbrock et al. 2015), osteoarthritis (Chang et al. 2019), and chronic pancreatitis (Staloch et al. 2015). Elevated *GREM1* mRNA has been identified in a wide range of human cancers including colorectal, breast, glioma, gastric and pancreatic cancer. These data and others are nicely summarised in some recent reviews (Elemam et al. 2022; Ouahoud et al. 2020; Todd et al. 2020). A germline gene duplication event upstream of the *GREM1* gene on chromosome 15 leads to amplified *GREM1* mRNA expression and hereditary mixed polyposis syndrome (HMPS) (Jaeger et al. 2012). These HMPS patients develop intestinal polyps and a colon cancer phenotype due to a tissue compartment switch in expression of *GREM1* from intestinal stromal to epithelial cells (Jaeger et al. 2012). Higher levels of *GREM1* also associate with poorer prognosis in patients with colorectal (Davis et al. 2015; Dutton et al. 2019a, b), gastric (Honma et al. 2018), and breast cancer (Neckmann et al. 2019) suggesting a role for *GREM1* as a tumor promotor. Many mechanistic studies link the pro-tumorigenic effect of *GREM1* to the promotion of a cancer stem-like and mesenchymal invasive phenotype and antagonism of BMP signaling in the stem cell niche, which facilitates stemness and self-renewal (Jaeger et al. 2012). For example, higher levels of *GREM1* were suggested to promote cancer stem cell (CSC) maintenance (Yan et al. 2014). Additionally, higher GREM1 expression in cancer-associated fibroblasts (CAFs) was postulated to create a favourable desmoplastic niche for the invasion of breast cancer cells (Fig. 1B) (Ren et al. 2019). However, GREM1-mediated responses that occur in other cells than CAFs, such as endothelial cells in the tumor stroma may also be significant for cancer development (Fig. 1B) (Gu et al. 2019). Moreover, these effects may occur in a manner independent of BMP antagonism (Mitola et al. 2010). Recent studies aimed at identifying non-BMP GREM1 binding partners and the characterization of the significance of these interactions in cancer and other diseases will be summarised below.

### GREM1 binding proteins and RTK activation

One of the first reports of a non-BMP binding partner for GREM1 in cancer cells was human tyrosine 3-monooxygenase/tryptophan 5-monooxygenase activation protein eta (14-3-3-eta, also called YWHAH,(Namkoong et al. 2006). The functional significance of this proposed interaction in currently unknown. GREM1 has also been shown to bind to heparan sulfate proteoglycan (HSPG) on the cell surface, via clusters of Arg and Lys residues along the exposed surface of the second β-strand finger loop in the C-terminus of GREM1 (Tatsinkam et al. 2015). These authors suggested that the binding of GREM1 to heparin and HSPGs could act to localise GREM1 to the extracellular matrix, creating a reservoir of GREM1 and spatially restrict it’s biological activity (Tatsinkam et al., 2015). In vivo, the interaction of GREM1 with HSPGs was shown to regulate both early and late phase neural crest induction (Pegge et al., 2020). While this GREM1-heparin/HSPG interaction was not required for GREM1-mediated antagonism of BMPs (Tatsinkam et al. 2017), GREM1 was reported to bind to secreted SLIT1 and SLIT2 extracellular matrix proteins that are ligands for Roundabout (ROBO) receptors. GREM interaction with SLIT was found to inhibit N-Formylmethionyl-leucyl-phenylalanine (fMLP) and stromal cell-derived factor (SDF)-1-induced monocyte activation (Chen et al. 2004). GREM1 binding may inhibit SLIT2-mediated activation of ROBO receptors in both neurons and kidney stem cells (Tumelty et al. 2018). Besides their important function in the formation of vascular networks, axon guidance and neuronal migration (Jiang et al. 2019), SLITs also play a role in cancer biology (e.g. Dickinson et al. 2004; Qiu et al. 2011; Yao et al. 2019; Rezniczek et al. 2019). However, no evidence of a role for GREM1-SLIT1/2 interaction in cancer has emerged to date.

A series of publications have demonstrated a novel role for GREM1 as ligand for the transmembrane vascular endothelial growth factor receptor (VEGFR)-2 in endothelial cells (Mitola et al. 2010) (Fig. 1A). The data suggested that GREM1 binding to VEGFR2 led to tyrosine receptor kinase activation and angiogenic responses in human umbilical vein endothelial cells (HUVECs) (Mitola et al. 2010). The interaction of GREM1 and VEGFR2 was analysed using recombinant GREM1 and the immobilised extracellular domain of VEGFR2 via surface plasmon resonance (SPR). Follow-up reports showed that both HSPGs and αvβ3 integrin were required for GREM1-mediated VEGFR2 activation and angiogenesis (Chiodelli et al. 2011; Ravelli et al. 2013). GREM1-mediated angiogenesis (potentially via VEGFR2 singalling) has been identified in pancreateic neuroendocrine tumors (Chen et al. 2013), colon cancer (Liu et al. 2019) and diffuse intrinsic pontine glioma (Shaik et al., 2017). In contrast to these agonistic effects of dimeric GREM1, the Mitola group and others found that monomeric GREM1 acted as an antagonist of VEGFR2 signaling in HUVECs and pulmonary microvascular endothelial cells (Grillo et al. 2016; Rowan et al. 2018). Of note, the Brazil group has cast doubt on the ability of GREM1 (up to 1 µg/ml, ~ 56 nM) to activate VEGFR2 in HUVECs or endothelial colony-forming cells (ECFCs), despite robust VEGF responses in these cells (Dutton et al. 2019a, b). BMPs have key roles in angiogenesis, and BMP and VEGF pathways display extensive crosstalk (Pulkkinen et al. 2021; Li et al. 2015; Deckers et al. 2002; Goumans et al. 2018). Therefore, GREM1-mediated antagonism of BMPs is also likely to contribute to the underlying mechanism of GREM1-regulated angiogenesis. GREM1 has also been reported to bind to and activate epidermal growth factor receptor (EGFR) in SKBR3 human breast cancer cells that overexpress HER2/EGFR (Park et al. 2020) (Fig. 1A). GREM1-mediated EGFR activation was suggested to enhance the promoter activity of estrogen-related receptor alpha (ERRα), which is predicted to bind to the *GREM1* promoter to increase GREM1 expression and drive breast cancer cell proliferation (Park et al. 2020). The interaction studies were performed by overexpressing EGFR or by incubating purified GREM1 with the EGFR extracellular domain (Park et al. 2020). This same group identified novel downstream signaling modalities for GREM1/EGFR activation, including STAT3/ERK/AKT activation and increased matrix metalloproteinase (MMP13) activity in breast cancer cells, leading to increased breast cancer cell invasion (Kim et al. 2020; Sung et al. 2020a, b).

In 2022, a new report from Cheng and colleagues revealed that GREM1 could bind to, and activate the fibroblast growth factor tyrosine kinase receptor 1 (FGFR1) (Cheng et al. 2022) (Fig. 1A). GREM1 binding to FGFR1 activated MEK/ERK signaling in prostate cancer cells, contributing to tumor progression and resistance to androgen deprivation (Cheng et al. 2022). Amino acids Lys147-Lys148 in the C-terminal region of GREM1 were identified as key residues for co-immunoprecipitation with FGFR1 (Cheng et al. 2022). GREM1 and FGF1 were found to interact with different domains in FGFR1, and were additive for FGFR1 activation (Cheng et al. 2022). A novel anti-GREM1 neutralizing antibody generated by this group demonstrated anti-tumor effects in prostate cancer cell-derived tumors in nude mice (Cheng et al. 2022). It is not clear from the published report if these neutralizing GREM1 antibodies affected GREM1-BMP binding. A demonstration of GREM1-mediated activation of FGFR1 in vivo (independent from its inhibitory effect on BMP signaling) in prostate cancer would strengthen the argument that GREM1 promotes prostate cancer progression by activating FGFR1. Given the lack of amino acid homology between GREM1 and FGF1/2 ligands, it will be interesting to explore whether GREM1-mediated FGFR1 signaling contributes to normal biological processes and/or other human tumor phenotypes.

## GREM1 as a tumor promotor and suppressor?

Many reviews have summarised the large volume of publications demonstrating elevated *GREM1* levels as a feature of diverse human tumors, where *GREM1* can be expressed in multiple cancer cells, but more frequently in CAFs. High levels of *GREM1* are also associated with poorer patient prognosis in, for example, colorectal and breast cancer (Davis et al. 2015; Dutton et al. 2019b; Neckmann et al. 2019), suggesting an oncogenic function for GREM1 in cancer. In contrast to the overwhelming consensus of GREM1 as a “bad actor” in human cancer, a small number of publications have presented data arguing the contrary. A report in 2010 identified *GREM1* as one of eight candidate tumor suppressor genes that displayed promoter-specific methylation in renal cell carcinoma (Fig. 1C) (Morris et al. 2010). Jang and colleagues identified that higher levels of *GREM1* mRNA were detected in stromal fibroblasts and were associated with improved recurrence-free CRC patient survival (Fig. 1D) (Jang et al. 2017). Chen and colleagues suggested that GREM1 is a prognostic marker for better survival in patients with pancreatic neuroendocrine tumors (NETs) (Fig. 1E) (Chen et al. 2013). Using immunohistochemistry staining and a polyclonal GREM1 antibody from Abnova, high GREM1 protein correlated with high microvessel density and well-differentiated NETs (Chen et al. 2013). These authors describe a tumor suppressor role for GREM1 in pancreatic NETs, although this conclusion relies solely on IHC data and not the more robust, quantifiable, and selective detection of *Grem1* mRNA. An ongoing issue in the field is the sensitivity and specificity of available GREM1 antibodies, and the absence of *Grem1*-/- tissue controls is a limitation of many studies.

As discussed above, Grem1 positivity in the tumor microenvironment is broadly associated with poor prognosis, reduced survival, and enhanced metastatic potential (“Bad” GREM1). A recent publication in Nature showed that in pancreatic adenocarcinoma (PDAC), the primary source of Grem1 expression is derived from tumor cells that have undergone epithelial-to-mesenchymal transition (EMT) and lost EpCAM expression (Lan et al. 2022). These Grem1-positive EMT-tumor cells reduced Snail and Slug expression in neighbouring EpCAM + cells, thereby maintaining their epithelial state (“Good GREM1”, Fig. 1F) (Lan et al. 2022). Loss of *Grem1* led to a rapid switch to a mesenchymal phenotype associated with enhanced metastasis in vivo, whereas Grem1 overexpression resulted in a less aggressive, primarily epithelial tumor phenotype (Lan et al. 2022). Interestingly, the genetic loss of *Grem1* in the tumor compartment reduced the abundance of myofibroblastic CAFs in the tumor microenvironment of their PDAC mouse model (Lan et al. 2022). Other studies showed that GREM1 overexpression in intestinal epithelial cells via the *Villin1* promotor protected mice from DSS-induced ulcer formation (Koppens et al. 2021) and subsequent restoration of Bmp signaling maintains epithelial cell integrity and differentiation (Ren et al. 2019; Li et al. 2019).

How can these apparently contradictory data be understood? One idea is that GREM1 may function differently in tumor cells compared to CAFs. Kapoor and colleagues identified that a Grem1 + fibroblast reticular cells in the stroma maintained dendritic cell homeostasis in lymphoid tissue, which may play an important role in some cancers (Kapoor et al. 2021). TGFβ-induced Grem1 expression in breast cancer CAFs promoted a mesenchymal phenotype, stemness and invasion of tumor cells (Ren et al. 2019). Consistently, siRNA-mediated targeting of *GREM1* in mesenchymal-like colon cancer cells (e.g. SW620) led to the suppression of cell growth and angiogenesis (Liu et al. 2019). To add to the complexity, CAFs can adopt a range of distinct functions in tumors. In various malignancies, CAFs have been identified with myofibroblastic, inflammatory and antigen-presenting functions (myofibloblast myCAFs, inflammatory iCAFs and antigen-presenting apCAFs, respectively) (Elyada et al. 2019; Costa et al. 2018; Sugimoto et al. 2006; Lenox and Bauer 2013). Alterations in the ratio of functionally distinct CAF subtypes in PDAC has previously been shown to differentially affect tumor progression. Inhibition of myCAF initiation and function in PDAC has previously been shown not to affect primary tumor growth, whereas inhibition of iCAFs reduced primary tumor growth (Biffi et al. 2019). Lan et al. reported more predominant *Grem1* expression levels in myCAFs than in iCAFs, and in vivo loss of *Grem1* within tumor cells reduced the amount of myCAFs, without affecting iCAF or apCAFs. We speculate that the observed altered ratio between myCAFs and iCAFs may result in a net increase in iCAFs and may affect the observed outcome (Lan et al. 2022).

The differential contribution of CAF subtypes to colorectal tumor progression has been elegantly summarised (Kobayashi et al. 2021). The authors showed the presence of *GREM1*-positive CAFs and immunoglobulin superfamily containing leucine-rich repeats (ISLR) (also known as Meflin)-positive CAFs in colorectal cancer. GREM1 and ISLR have opposing effects on BMP signaling, with GREM1 acting as an inhibitor and ISLR acting as a stimulator of BMP signaling. Whereas high Grem1 expression was correlated with reduced disease-free survival, high Islr correlated with improved survival. Grem1-positive CAFs appeared to be transforming growth factor beta (TGFβ)-responsive myofibroblastic CAFs based on their co-expression with smooth muscle actin (SMA) and promotion of colorectal tumoroid growth. In contrast, the ISLR-positive CAFs appeared to have tumor-inhibiting effects in vitro and in vivo (Kobayashi et al. 2021). These data clearly show that differential regulation of BMP signaling induced by stromal cells affects tumor cell behavior and highlights the delicate balance between pro- and anti-tumorigenic CAFs. Other groups identified increased *Grem1* mRNA expression in myCAFs in basal cell carcinoma, suggesting that this hypothesis extends beyond colorectal cancer (Kim et al. 2017).

A few publications have suggested that increased GREM1 expression may increase pro-inflammatory cytokine expression, contributing to a chronic inflammatory environment (Han et al. 2016). Inflammatory conditions such as osteoarthritis (Han et al. 2016; Chang et al. 2019) and renal inflammation (Lavoz et al. 2015; Lavoz et al. 2018) and damage are promoted by GREM1 overexpression, potentially via VEGFR2 signaling (Lavoz et al. 2015). GREM1-mediated CREB and NFkB signaling has been proposed to increase the expression of pro-inflammatory chemokines such as CCL2 and adhesion molecules such as ICAM1 (Corsini et al. 2014). Many of these effects of Grem1 are likely to be tissue dependent. In mouse models of lung fibrosis, inflammatory fibroblasts expressing Grem1 play a key role at inducing the early pathological steps changes associated with the onset of fibrosis (Li et al. 2022). Furthermore, studies from three groups (Koppens et al. 2021; Cox et al., 2021; Goto et al. 2022) highlighted the importance of *Grem1*-positive fibroblasts in intestinal regeneration and stemness. Interleukin (IL)-1R1 signaling in *Grem1* + mesenchymal cells led to the production of the Wnt agonist R-spondin, which was required for intestinal stem cell self-renewal (Cox et al. 2021). Although these studies were performed under conditions of injury rather than cancer, one could hypothesize that similar functions of *Grem1*^+^ fibroblasts/mesenchymal cells in a cancer setting create a favourable microenvironment in which tumor progression is enhanced by increasing stemness.

## Future perspectives

“Signatures of all things I am here to read” said Stephen Dedalus in the Proteus chapter of Ulysses by James Joyce. Despite the overwhelming number of reports in the literature supporting a tumor-promoting role for GREM1 in a range of cancers, the small number of reports describing the tumor-suppressing role of GREM1 must also be carefully considered. Some reports identified non-BMP dependent GREM1 signaling activity in a range of cancer cells (e.g.(Sung et al. 2020a, b; Chang et al. 2019). However, we suggest that conclusions based solely on in vitro protein-protein interaction involving molar excesses of recombinant proteins need to be cautiously made. It is critical that the commercial sources of rhGREM1 used in different papers are compared and analysed e.g. by mass spectrometry to identify potential contaminant proteins that may influence results. Demonstration of the interaction of endogenously expressed GREM1 with EGFR and FGFR1 in vivo would significantly increase confidence in the true physiological (or pathophysiological) nature of these interactions. Highly selective gene deletion of GREM1 using CRISPR/Cas9 techniques, or its predicted binding partners will address some of the issues regarding the pathophysiological significance of these interactions. However, care needs to be taken in interpreting data from transgenic/knockout mice using diverse CRE recombinase constructs and purported tissue-specific promotors that may lead to off-target effects (Gooding and Leedham 2020). Moreover, CRISPR/Cas9 techniques can be used to epitope tag GREM1 or its binding partners and allow for endogenous protein interaction studies, for example using sensitive BioID proximity labelling methods in which the bait protein is fused to a promiscuous biotin ligase (Rosenthal et al. 2021). Delineation of the BMP *versus* non-BMP binding functions of GREM1 is also critical. A GREM1 K147A/K148A mutant generated by Cheng et al. was shown to bind less well to FGFR1 compared to wild-type GREM1 (Cheng et al. 2022). It remains to be determined if this GREM1 mutant retains the ability to bind BMPs. If it does, then GREM1 inhibition of BMP function and activation of FGFR1 could be uncoupled. Similarly, the corollary question can be formulated to assess whether GREM1 mutants that have lost their BMP binding capacity can still activate FGFR1, EGR and other RTKs yet to be identified. This may be achieved using CRISPR/Cas9 mediated base-editing at the endogenous locus of GREM1 or FGFR1. By extension, the generation of transgenic mice expressing these and other mutants will help us dissect the intricacies of GREM1 signaling in vivo. These novel mouse mutants could also be challenged with various cancer models to determine if the expression of various *Grem1* mutants has any effect on tumor formation and cancer progression. The availability of GREM1 neutralising antibodies that specifically inhibit interaction with BMP or FGFR1 would be highly informative to elucidate the importance of GREM1 in pathophysiological contexts. Side-by-side analysis of antibodies that block Grem1-Bmp versus Grem1-Fgfr1 interactions in the prostate cancer mouse model would be a useful approach to help delineate the relative contribution of canonical (Bmp) versus non-canonical (Fgfr1) Grem1 signaling in this model. Importantly, anti-GREM1 antibodies from several companies are in development and may be useful tools to address this question. A group from Novartis detailed the potential of a GREM1 antibody that interferes with Grem1-Bmp interaction in a mouse model of pulmonary artery hypertension (Ciuclan et al. 2013). Regeneron have also patented a GREM1 antibody blocking GREM1-BMP binding in 2019 to be used to treat the symptoms of fibrosis in the liver, lungs, or kidneys (https://patents.google.com/patent/US20160024195A1/en). Intra-articular injection of this GREM1 antibody decelerated osteoarthritis in a mouse model (Chang et al. 2019). A Korean group has developed a GREM1 antibody that can bind to and inhibit GREM1 function independent of BMP and VEGFR2 (https://patents.google.com/patent/KR20210028191A/en?q=GREM1+korea+antibody&oq=GREM1&korea+antibody). A report in 2012 suggested that this anti-GREM1 antibody could reduce GREM1-mediated A549 lung cancer cell proliferation, invasion, and migration (Kim et al. 2012). UCB has developed a human IgG4p monoclonal antibody called Ginisortamab that targets endogenous GREM1 and has been shown to neutralise its ability to antagonise BMP2 (Kobayashi et al. 2021). Ginisortamab has been shown to have anti-tumor effects in pre-clinical murine models of colorectal cancer (Kobayashi et al. 2021) and multiple myeloma (Clark et al. 2020). Transcenta have developed a monoclonal GREM1 antibody (TST003) that could inhibit GREM1-mediated activation of FGFR1 phosphorylation (Cheng et al. 2022). At this stage, it is not clear whether the UCB or Transcenta antibodies selectively inhibit GREM1 binding to BMPs and FGFR1 respectively. Ginistortamab is currently in a phase 1/2 open-label study to assess its safety, pharmacokinetics, and anti-tumor activity in patients with advanced solid tumors, including gastrointestinal tumours.(https://classic.clinicaltrials.gov/ct2/show/NCT04393298?term=UCB6114&draw=2&rank=1) (https://ascopubs.org/doi/10.1200/JCO.2022.40.4_suppl.TPS221). TST003 from Transcenta received IND approval from the FDA in September 2022 for the treatment of prostate and other cancers (https://www.transcenta.com/newsDet/id-98). The results from these clinical trials should give us the first indication of the potential of targeting GREM1 in GI and other cancers in humans. (https://clinicaltrials.gov/ct2/show/NCT04393298?term=UCB6114&draw=2&rank=1) (https://ascopubs.org/doi/abs/10.1200/JCO.2022.40.4_suppl.TPS221).

## Conclusions

Despite an overwhelming volume of publications suggesting that high levels of GREM1 contribute to cancer progression and worsening patient prognosis, the report from Lan and colleagues discussed above (Lan et al. 2022) and the apparent disparity around the oncogenic *versus* tumor suppressor role of GREM1 in pancreatic (and other) cancer must be carefully considered. Delivery of Grem1 neutralising antibodies that inhibit Grem1 binding to target Bmps to in vivo mouse models of pancreatic cancer would allow investigators to assess whether this leads to improved or worsened tumor volume and outcomes. A recent commentary alludes to “the usefulness of agonistic GREMLIN-like biologicals that could possibly mediate differentiation therapy in human tumors” (Moustakas et al. 2022). Given the divergent reports and conclusions about the true pathophysiological role of GREM1 in different human cancers, we would suggest caution in this approach, as any agent that increases the tone or volume of GREM1 signaling may exacerbate the cancer phenotype and potentially worsen patient outcomes (for example, see Jaeger et al. 2012). More work is needed to unravel the ineluctable complexity of GREM1 biology and signaling in human health and disease. We look forward to emerging reports and data that will shed “bright light” on our understanding of GREM1 biology, providing a clearer path forward for the potential of GREM1 targeting in human disease.
